# Molecular and Clinical Characterization of LAG3 in Breast Cancer Through 2994 Samples

**DOI:** 10.3389/fimmu.2021.599207

**Published:** 2021-06-29

**Authors:** Qiang Liu, Yihang Qi, Jie Zhai, Xiangyi Kong, Xiangyu Wang, Zhongzhao Wang, Yi Fang, Jing Wang

**Affiliations:** Department of Breast Surgical Oncology, National Cancer Center/National Clinical Research Center for Cancer/Cancer Hospital, Chinese Academy of Medical Sciences and Peking Union Medical College, Beijing, China

**Keywords:** cancer immunotherapy, CD223, LAG3, immune response, inflammatory activity

## Abstract

Despite the promising impact of cancer immunotherapy targeting CTLA4 and PD1/PDL1, numerous cancer patients fail to respond. LAG3 (Lymphocyte Activating 3), also named CD233, serves as an alternative inhibitory receptor to be targeted in the clinic. The impacts of LAG3 on immune cell populations and coregulation of immune responses in breast cancer remain largely unknown. To characterize the role of LAG3 in breast cancer, we investigated transcriptome data and associated clinical information derived from 2,994 breast cancer patients. We estimated the landscape of the relationship between LAG3 and 10 types of cell populations of breast cancer. We investigated the correlation pattern between LAG3 and immune modulators in pancancer, particularly the synergistic role of LAG3 with other immune checkpoint members in breast cancer. LAG3 expression was closely related to the malignancy of breast cancer and may serve as a potential biomarker. LAG3 may play an important role in regulating the tumor immune microenvironment of T cells and other immune cells. More important, LAG3 may synergize with CTLA4, PD1/PDL1, and other immune checkpoints, thereby contributing more evidence to improve combination cancer immunotherapy by simultaneously targeting LAG3, PD1/PDL1, and CTLA4.

## Introduction

Breast cancer is the most common malignancy and the leading cause of death of women worldwide (1). Despite significant progress in comprehensive therapy, such as breast conservation surgery/radical mastectomy, neoadjuvant/adjuvant chemotherapy, adjuvant radiotherapy, targeted therapy, endocrine therapy, and emerging immunotherapy, approximately 46 million women die from breast cancer each year (2). Patients who suffer from recurrence and metastasis of breast cancer have a relatively short median survival time due to the aggressiveness of tumors, the low response rate to immunotherapy, and resistance to treatment (2).

In the past decade, many studies focused on the immunotherapy of various cancers, which show the benefits from inhibiting the interaction between programmed death-1 (PD-1) and its ligand-1 (PD-L1) to inhibit the suppression of T cell immune responses (3). Meanwhile, several clinical trials of PD-1/PD-L1 targeting breast cancer were initiated (4). However, given that the objective response rates range between 13 and 56% and complete response rates range between 1 and 16%, the success of such emerging therapy is limited, particularly for breast cancer (5–11). Therefore, the efficacy and mechanism of immunotherapy is not fully understood, and more research is needed.

Numerous studies discovered several negative costimulatory molecules such as the programmed death 1 (PD-1)/programmed death ligand 1 (PD-L1) axis, lymphocyte activation gene-3 (LAG3), cytotoxic T lymphocyte associated antigen-4 (CTLA4), T-cell immunoglobulin and mucin domain protein 3 (TIM3), which participate to inhibit T cells and enable different tumor cells to singly or jointly escape (3, 12–14).

Overexpression of inhibitory receptors (IRs) is significant to balance costimulatory receptor activity and to limit T-cell activation, thus helping to prevent autoimmunity, autoinflammation, and tissue damage. Despite the impressive impact of CTLA4 and PD1-PDL1-targeted cancer immunotherapy, LAG3 (also named CD223), serving as a cancer immunotherapy target, is the third IR to be targeted in the clinic due to its negative regulatory role for T cells and its capacity, combined with PD1, to mediate a state of exhaustion (15), consequently attracting considerable interest and scrutiny (12). LAG3 belongs to the Ig superfamily and contains four extracellular Ig-like domains. LAG3 is highly expressed by activated human T and NK cells, as well as by tumor infiltrating lymphocytes (TILs), in various tumors. Previous studies show that as an inhibitory receptor on antigen activated T-cells, LAG3 delivers T cell inhibitory signals upon binding to ligands such as FGL1 (by similarity) (16–18).

LAG3 was suggested to be spatially associated with the T-cell receptor (TCR), particularly with CD3-TCR, in the immunological synapse and to directly inhibit T-cell activation (by similarity) (12). Furthermore, LAG3 negatively regulates the activation, proliferation, homeostasis, and effector function of CD4(+) and CD8(+) T cells. Moreover, immune tolerance is mediated by LAG3, which is constitutively expressed by a subset of regulatory T-cells (Tregs), consequently contributes to their suppressive function (by similarity) (16–18). LAG3 is involved as well in inhibiting antigen-specific T-cell activation in synergy with PDCD1/PD-1, which possibly acts as a coreceptor for PDCD1/PD-1 (by similarity) and in influencing the therapeutic effect of blocking one of them (12). LAG3 acts as a negative regulator of plasmacytoid dendritic cell (pDCs) activation (by similarity) with the potential to bind MHC class II (MHC-II), although the precise role of MHC-II-binding is unclear (12).

Previous studies show that LAG3 suppresses T cell activation and antitumor responses *in vitro* (19–21). However, they do not show the specific expression pattern of LAG3 and its potential impact on other immune cell populations and immune modulators. In the present study, we systematically investigated the LAG3-related transcriptome profile to reveal its potential role in inducing immune responses and inflammatory activities, as well as its potential relationship with immune modulators. This study is the first integrative analysis, to our knowledge, to molecularly and clinically characterize the landscape of LAG3 expression in breast cancer.

## Materials and Methods

### Data Collection

TCGA dataset was downloaded through GDCRNATools (access date: Feb 01, 2020) (22). Raw counts data were normalized through TMM implemented in edgeR (23) and were then transformed by voom in limma (24); and only genes with cpm > 1 in more than half of samples were kept. Selected TCGA breast cancer clinical data were kindly provided by Dr. Hai Hu and Dr. Jianfang Liu of the Chan Soon-Shiong Institute of Molecular Medicine at Windber. HER2 status was determined using DNA copy numbers for cases without IHC or FISH status. Standardized survival data were retrieved from TCGA Pan-Cancer Clinical Data Resource (TCGA-CDR) (25). The METABRIC dataset (26) containing 1,904 cases was retrieved from the cBioPortal database (access date: Feb 01, 2019).

### Bioinformatics Analysis

The biological functions of the genes correlated with LAG3 were analyzed using the clusterProfiler package (27). GO terms and KEGG pathways with adjusted P values <0.05 were considered significant. Immunologically related genes were collected from the Immunology Database and Analysis Portal (ImmPort) (28). The absolute abundances of eight immune and two stromal cell populations were determined using Microenvironment Cell Populations-counter method (29). Gene set variation analysis (GSVA) analysis (30) was performed to estimate the abundance of GO gene sets related to specific immune functions and inflammatory metagenes (31). Correlations between LAG3 and immune modulators in pancancer were analyzed using the TISIDB database (32), an integrated repository portal for tumor-immune system interactions. Spearman correlation analyses were performed to evaluate the correlations between LAG3 and metagenes and specific immune functions.

### Statistical Analysis

Correlations between continuous variables were assessed using Spearman correlation analyses. Differences in variables between groups were evaluated through the Student *t* test, one-way ANOVA, or the Pearson’s chi-squared test. All statistical tests were performed using R (version 3.6.0; https://www.r-project.org/). Other statistical calculations and graphical representations were performed using ggplot2 (33), pheatmap, pROC (34), circlize (35), and corrgram (36). P < 0.05 was considered significant. All statistical tests were two-sided.

## Results

### Associations of LAG3 Expression With Clinical and Molecular Characteristics of Breast Cancer

To characterize the association between LAG3 expression and clinical characteristics of breast cancer patients, we dichotomized patients into low- and high-expression groups according to the median cut-off value of LAG3 expression. Associations of LAG3 expression and clinical characteristics in TCGA (n = 1,090) and METABRIC cohorts (n = 1,904) are listed in **Tables 1**, **2**. LAG3 was associated with AJCC stage, ER, PR, and HER2 status in both datasets, and was associated with T stage in TGCA data, as well as age, tumor size, and tumor grade in the METABRIC cohort. We further explored the expression patterns of LAG3 associated with molecular and clinical characteristics.

**Table 1:** Association Between LAG3 mRNA Expression and Clinicopathologic Characteristics in TCGA Cohort.

	Expression			
	Total (n = 1,090)	LAG3 high (n = 545)	LAG3 low (n = 545)	P-value
**Age (years)**				
>=55	517 (47.4%)	258 (47.3%)	259 (47.5%)	0.952
<55	573 (52.6%)	287 (52.7%)	286 (52.5%)	
**T stage**				
T1	279 (25.6%)	118 (21.7%)	161 (29.5%)	0.016
T2	631 (57.9%)	339 (62.2%)	292 (53.6%)	
T3	137 (12.6%)	71 (13.0%)	66 (12.1%)	
T4	40 (3.7%)	16 (2.9%)	24 (4.4%)	
Missing	3 (0.3%)	1 (0.2%)	2 (0.4%)	
**N stage**				
N0	514 (47.2%)	256 (47.0%)	258 (47.3%)	0.114
N1	360 (33.0%)	172 (31.6%)	188 (34.5%)	
N2	120 (11.0%)	67 (12.3%)	53 (9.7%)	
N3	76 (7.0%)	44 (8.1%)	32 (5.9%)	
Missing	20 (1.8%)	6 (1.1%)	14 (2.6%)	
**M stage**				
M0	907 (83.2%)	447 (82.0%)	460 (84.4%)	0.414
M1	22 (2.0%)	10 (1.8%)	12 (2.2%)	
Unknown	161 (14.8%)	88 (16.1%)	73 (13.4%)	
**AJCC stage**				
I	181 (16.6%)	72 (13.2%)	109 (20.0%)	0.027
II	621 (57.0%)	324 (59.4%)	297 (54.5%)	
III	250 (22.9%)	133 (24.4%)	117 (21.5%)	
IV	20 (1.8%)	9 (1.7%)	11 (2.0%)	
Missing	18 (1.7%)	7 (1.3%)	11 (2.0%)	
**ER status**				
Negative	236 (21.7%)	169 (31.0%)	67 (12.3%)	<0.001
Positive	803 (73.7%)	355 (65.1%)	448 (82.2%)	
Unknown	51 (4.7%)	21 (3.9%)	30 (5.5%)	
**PR status**				
Negative	343 (31.5%)	221 (40.6%)	122 (22.4%)	<0.001
Positive	694 (63.7%)	302 (55.4%)	392 (71.9%)	
Unknown	53 (4.9%)	22 (4.0%)	31 (5.7%)	
**HER2 status**				
Negative	895 (82.1%)	438 (80.4%)	457 (83.9%)	0.006
Positive	168 (15.4%)	99 (18.2%)	69 (12.7%)	
Unknown	27 (2.5%)	8 (1.5%)	19 (3.5%)	

**Table 2:** Association Between LAG3 mRNA Expression and Clinicopathologic Characteristics in METABRIC Cohort.

	Expression			
	Total(n = 1,904)	LAG3 high(n = 952)	LAG3 low(n = 952)	P-value
**Age (years)**				
>=55	952 (50.0%)	511 (53.7%)	441 (46.3%)	<0.001
<55	952 (50.0%)	441 (46.3%)	511 (53.7%)	
**Tumor size**				
>=2 cm	592 (31.1%)	268 (28.2%)	324 (34.0%)	0.021
<2 cm	1,292 (67.9%)	673 (70.7%)	619 (65.0%)	
Missing	20 (1.1%)	11 (1.2%)	9 (0.9%)	
**AJCC stage**				
0	4 (0.2%)	1 (0.1%)	3 (0.3%)	0.003
I	475 (24.9%)	209 (22.0%)	266 (27.9%)	
II	800 (42.0%)	426 (44.7%)	374 (39.3%)	
III	115 (6.0%)	69 (7.2%)	46 (4.8%)	
IV	9 (0.5%)	3 (0.3%)	6 (0.6%)	
Missing	501 (26.3%)	244 (25.6%)	257 (27.0%)	
**Tumor Grade**				
I	165 (8.7%)	31 (3.3%)	134 (14.1%)	<0.001
II	740 (38.9%)	288 (30.3%)	452 (47.5%)	
III	927 (48.7%)	598 (62.8%)	329 (34.6%)	
Missing	72 (3.8%)	35 (3.7%)	37 (3.9%)	
**ER status**				
Negative	445 (23.4%)	334 (35.1%)	111 (11.7%)	<0.001
Positive	1,459 (76.6%)	618 (64.9%)	841 (88.3%)	
**PR status**				
Negative	895 (47.0%)	568 (59.7%)	327 (34.3%)	<0.001
Positive	1,009 (53.0%)	384 (40.3%)	625 (65.7%)	
**HER2 status**				
Negative	1,668 (87.6%)	786 (82.6%)	882 (92.6%)	<0.001
Positive	236 (12.4%)	166 (17.4%)	70 (7.4%)	

**Figure 1:**
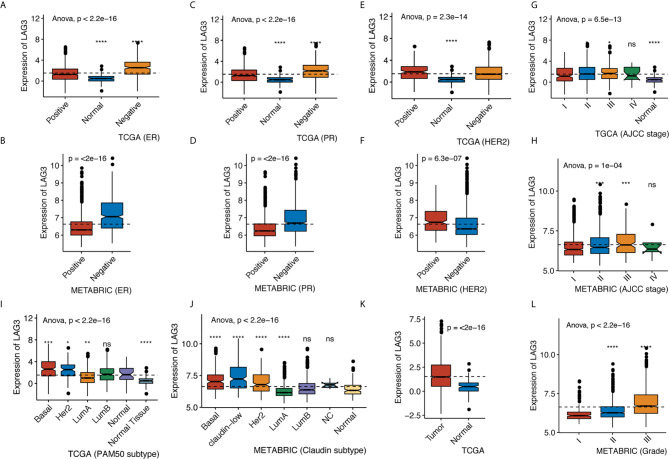
LAG3 expression classified by ER status in TCGA **(A)** and METABRIC **(B)**, by PR status in TCGA **(C)** and METABRIC **(D)**, by HER2 status in TCGA **(E)** and METABRIC **(F)**, by AJCC stage in TCGA **(G)** and METABRIC **(H)**, by PAM50 subtype in TCGA **(I)** and Claudin subtype in METABRIC **(J)**, by tumor diagnosis in TCGA **(K)** and by grade in METABRIC **(L)**. (*P < 0.05, **P < 0.01, ***P < 0.001, ***P < 0.0001).

We found that LAG3 was upregulated in breast cancer tissues compared with normal tissues in TCGA data (**Figure 1K**). We found that LAG3 expression was upregulated in the ER-negative and PR-negative groups in the TCGA and METABRIC databases (**Figures 1A–D**) as well as in the HER2-negative group of the METABRIC database, but this was not observed using TCGA data (**Figures 1E, F**). LAG3 was overexpressed in higher tumor stages compared with stage I, although not significant in stage IV (**Figures 1G, H**). We found that LAG3 was enriched in the basal, HER2-positive, and luminal A (LumA) subtypes, but not in the luminal B (LumB) subtype. These results were mutually validated using TCGA and METABRIC data.

**Figure 2:**
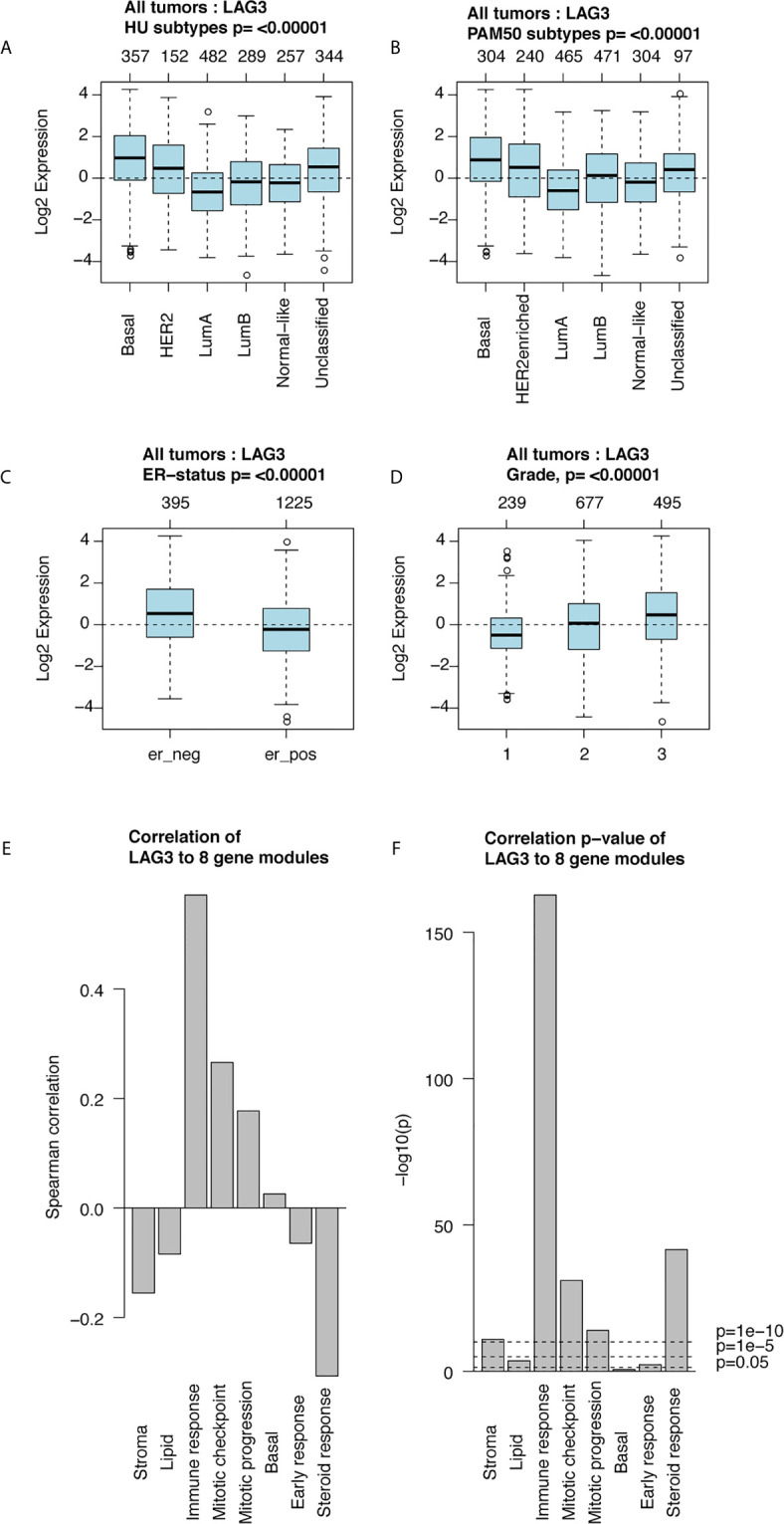
LAG3 expression in 1,881-sample dataset. LAG3 expression across different subtypes and tumor stage **(A–D)**; Correlation of LAG3 and gene modules **(E, F)**.

Furthermore, we found higher expression of LAG3 in association with higher tumor grades (**Figure 1L**). These results were further validated using independent microarray datasets derived from the GOBO database (n = 1,881) (37); and correlation analysis revealed that LAG3 expression strongly correlated with immune response gene modules, which suggests that they play important roles in immune-related functions (**Figures 2A–F**). In summary, these findings indicate that high expression of LAG3 predicted highly malignant breast cancer.

### LAG3 Is a Potential Biomarker for the TNBC Subtype

To further explore the association of LAG3 expression and the malignancy of breast cancer, we compared the expression of LAG3 between the TNBC and None-TNBC groups. We found that LAG3 was significantly upregulated in the TNBC group of the TCGA (n = 1,090) and METABRIC (n = 1,904) databases (**Figures S1A, B**). To further validate these findings, ROC curve analyses of LAG3 expression and the TNBC subtype of all breast cancers were performed. Our results, indicated by the area under the curve (AUC), were up to 0.707 and 0.726 in TCGA and METABRIC datasets, respectively (**Figures S1C, D**). These findings suggest that LAG3 plays a pivotal role in the progression of breast cancer. Moreover, LAG3 may serve as a potential biomarker for TNBC.

### LAG3 Is Closely Related to Immune Functions in Breast Cancer

**Figure 3:**
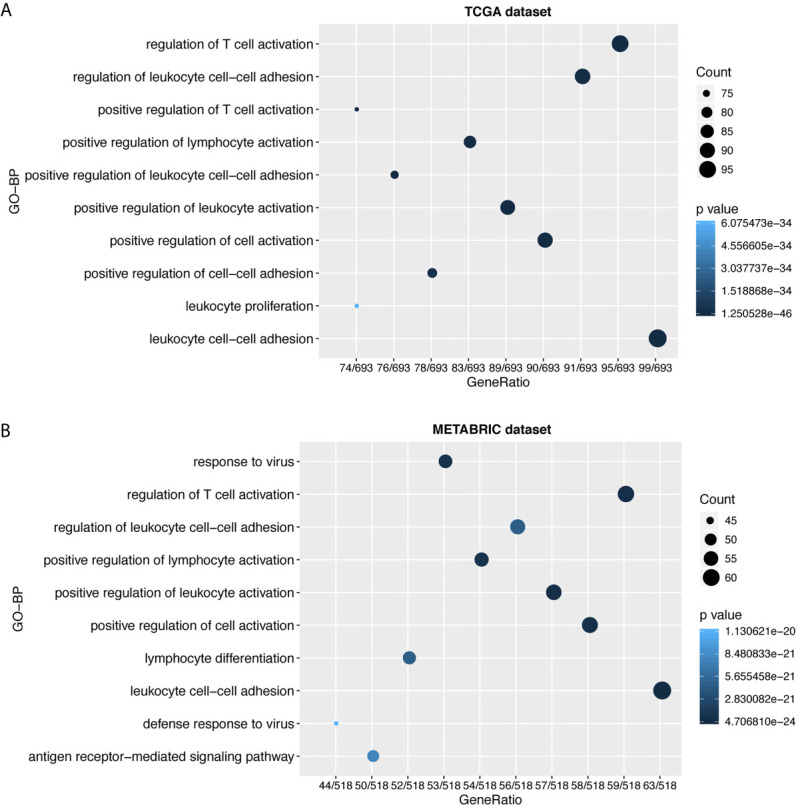
LAG3 was closely related to immune functions in breast cancer. Gene ontology analysis showed that LAG3 was mainly involved in immune response and inflammatory response in TCGA and METABRIC databases **(A, B)**.

**Figure 4:**
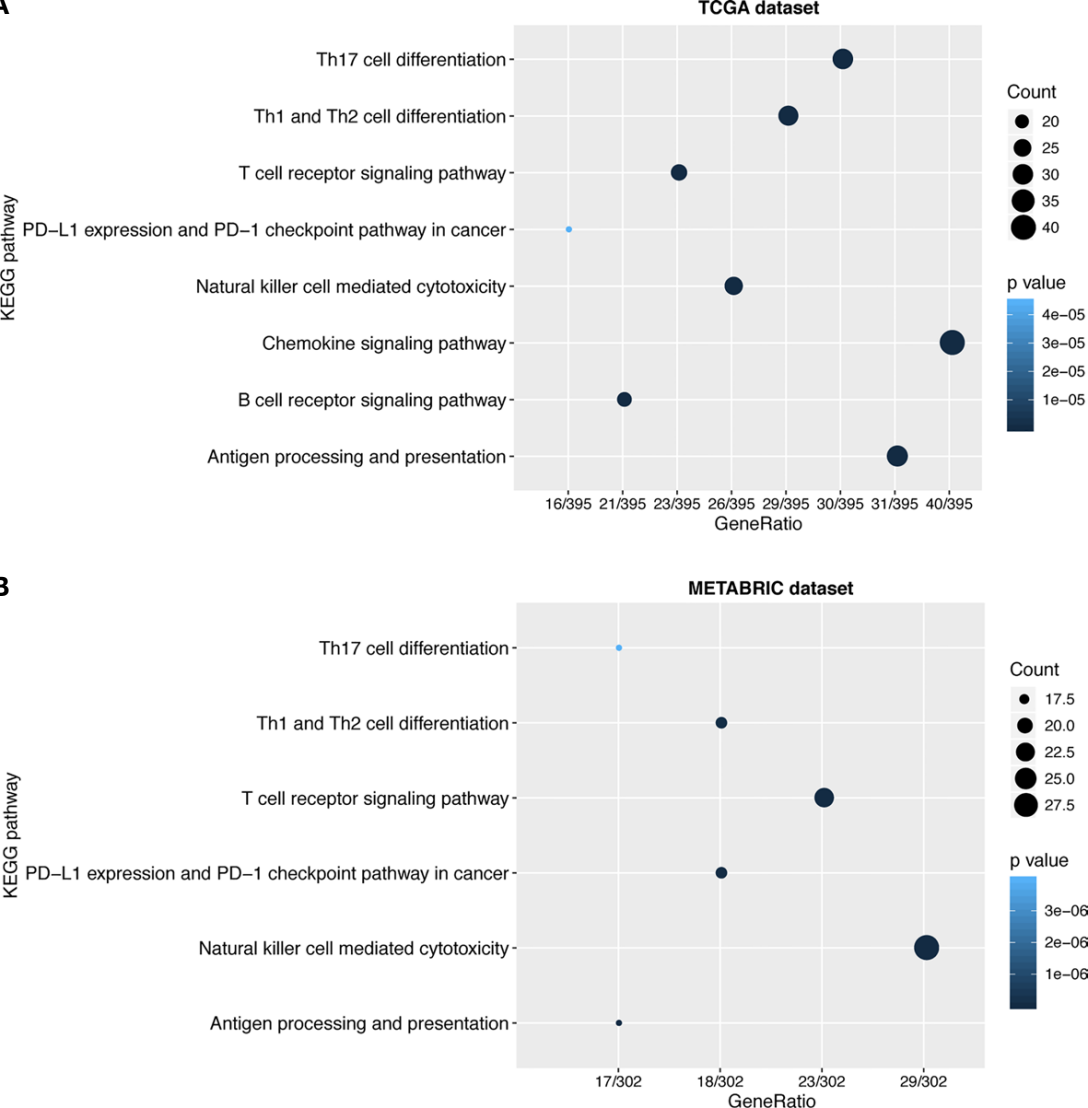
LAG3 was closely related to immune cell related signaling pathways. KEGG analysis revealed LAG3 was involved in T cell related signaling pathways, B cell related pathways, and immune checkpoint related pathways **(A, B)**.

To further explore the biological functions of LAG3 in breast cancer, we screened 746 and 582 genes that strongly correlated with LAG3 according to Spearman correlation analyses (|R| > 0.4 and P < 0.05) of the TCGA and METABRIC datasets, respectively. Subsequently, GO and KEGG functional enrichment analyses were performed to understand the biological roles of LAG3. Consistent with the aforementioned results derived from a 1,881-sample microarray dataset, GO analyses revealed that genes correlated with LAG3 were mainly enriched in biological processes related to immune response and inflammatory activities, particularly in the regulation of T cells, leukocytes, and lymphocytes; and these results were mutually validated using the TCGA and METABRIC datasets (**Figures 3A, B**). Furthermore, KEGG analysis revealed that LAG3-related genes were enriched in pathways related to T cells, PD-L1 expression, and PD-1 checkpoint pathways in cancer, natural killer cell-mediated cytotoxicity, and antigen processing and presentation in TCGA and METABRIC datasets (**Figures 4A, B**). These findings further indicate the important role of LAG3 in mediating immune-related functions during breast cancer progression.

### LAG3-Related Immune Response

**Figure 5:**
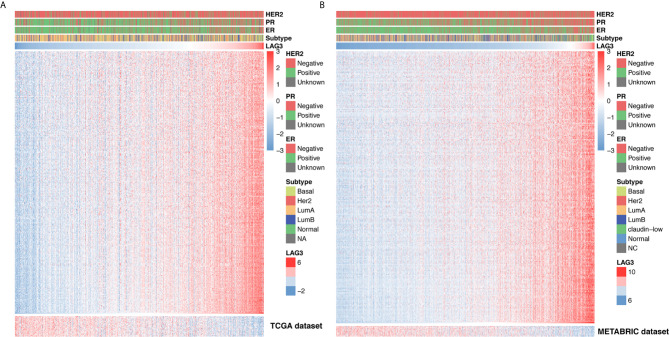
LAG3 related immune responses. Most immune-related genes were positively correlated with LAG3 expression in TCGA and METABRIC databases, while a small number of genes were negatively associated **(A, B)**.

To further clarify the role of LAG3 in the immune response to breast cancer, we collected 4,723 immunologically related genes from The Immunology Database and Analysis Portal (ImmPort) database. We selected genes that were most relevant to LAG3 (Spearman |R| > 0.4, P < 0.05) to draw the heatmaps. We found that 322 and 254 immunologically related genes positively correlated with LAG3 in TCGA and METABRIC databases, respectively, and only 25 and 10 immunologically related genes negatively correlated with LAG3, respectively (**Figures 5A, B**). These results indicate that LAG3 positively correlated with most relevant immune responses and negatively correlated with a small number of immune responses to breast cancer.

### Association of LAG3 Expression and Immune Cell Populations

**Figure 6:**
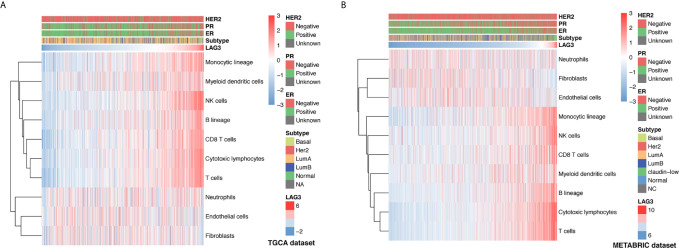
Association between LAG3 expression and immune cell populations in TCGA **(A)** and METABRIC **(B)** databases.

To further understand the immune regulatory role of LAG3 in breast cancer, we estimated the absolute abundance of eight immune and two stromal cell populations from transcriptome data through the Microenvironment Cell Populations-counter method (29). Interestingly, we observed that LAG3 expression positively correlated with T cells, CD8 T cells, cytotoxic lymphocytes, NK cells, B cell lineages, the monocytic lineage, and myeloid dendritic cells, but not neutrophils, endothelial cells, and fibroblasts (**Figures 6A, B**). LAG had the strongest correlation with T cells, indicating the important role of LAG3 in T cell-induced immune functions in breast cancer. The detailed correlation coefficients between LAG3 and aforementioned cell abundances are listed in **Table 3**. These results were mutually verified using the TCGA and METABRIC datasets.

**Table 3:** Association Between LAG3 mRNA Expression and Immune Cell Populations in TCGA and METABRIC Databases.

	Gene	METABRIC		TCGA	
		rho	P-value	rho	P-value
rho	T cells	0.71	<0.001	0.71	<0.001
rho1	CD8 T cells	0.39	<0.001	0.65	<0.001
rho2	Cytotoxic lymphocytes	0.70	<0.001	0.63	<0.001
rho3	NK cells	0.43	<0.001	0.64	<0.001
rho4	B lineage	0.52	<0.001	0.48	<0.001
rho5	Monocytic lineage	0.58	<0.001	0.61	<0.001
rho6	Myeloid dendritic cells	0.28	<0.001	0.38	<0.001
rho7	Neutrophils	−0.08	<0.001	0.12	<0.001
rho8	Endothelial cells	−0.09	<0.001	−0.08	0.006
rho9	Fibroblasts	−0.19	<0.001	−0.07	0.014

### The Relationship Between LAG3 Expression and Immune Modulators in Pancancer

**Figure 7:**
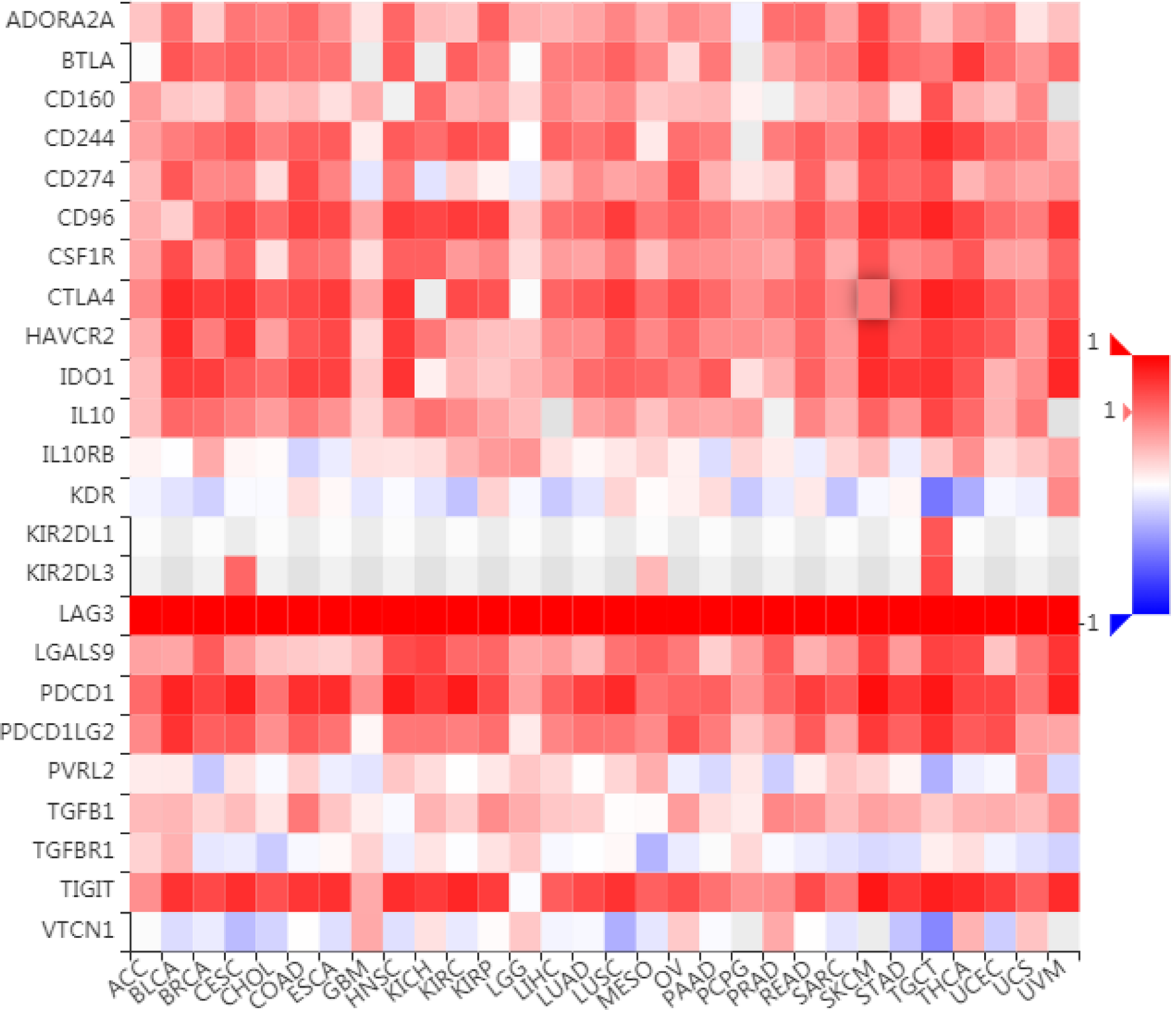
LAG3 expression is correlated with immunoinhibitors in pan-cancer.

**Figure 8:**
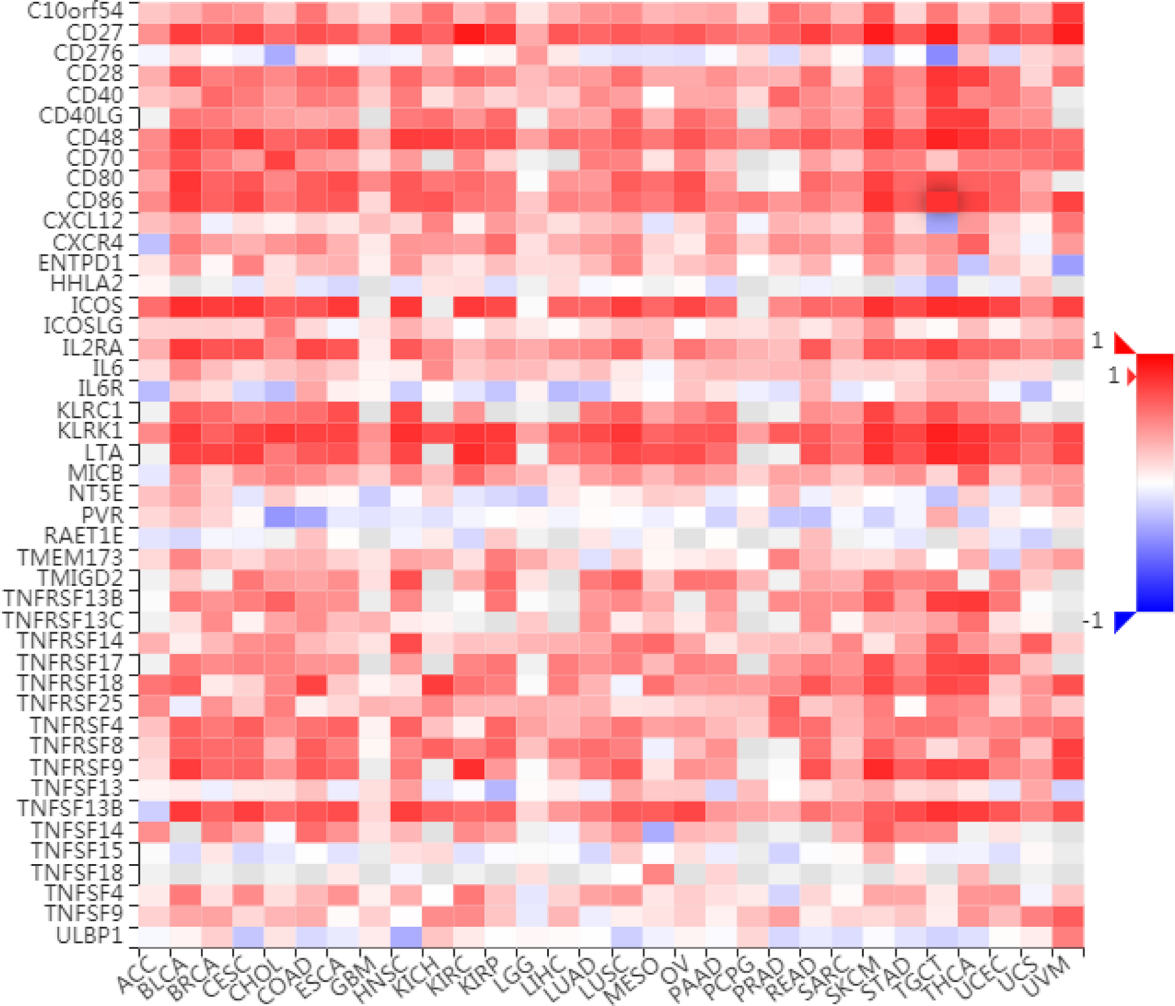
LAG3 expression is correlated with immunostimulators in pan-cancer.

**Figure 9:**
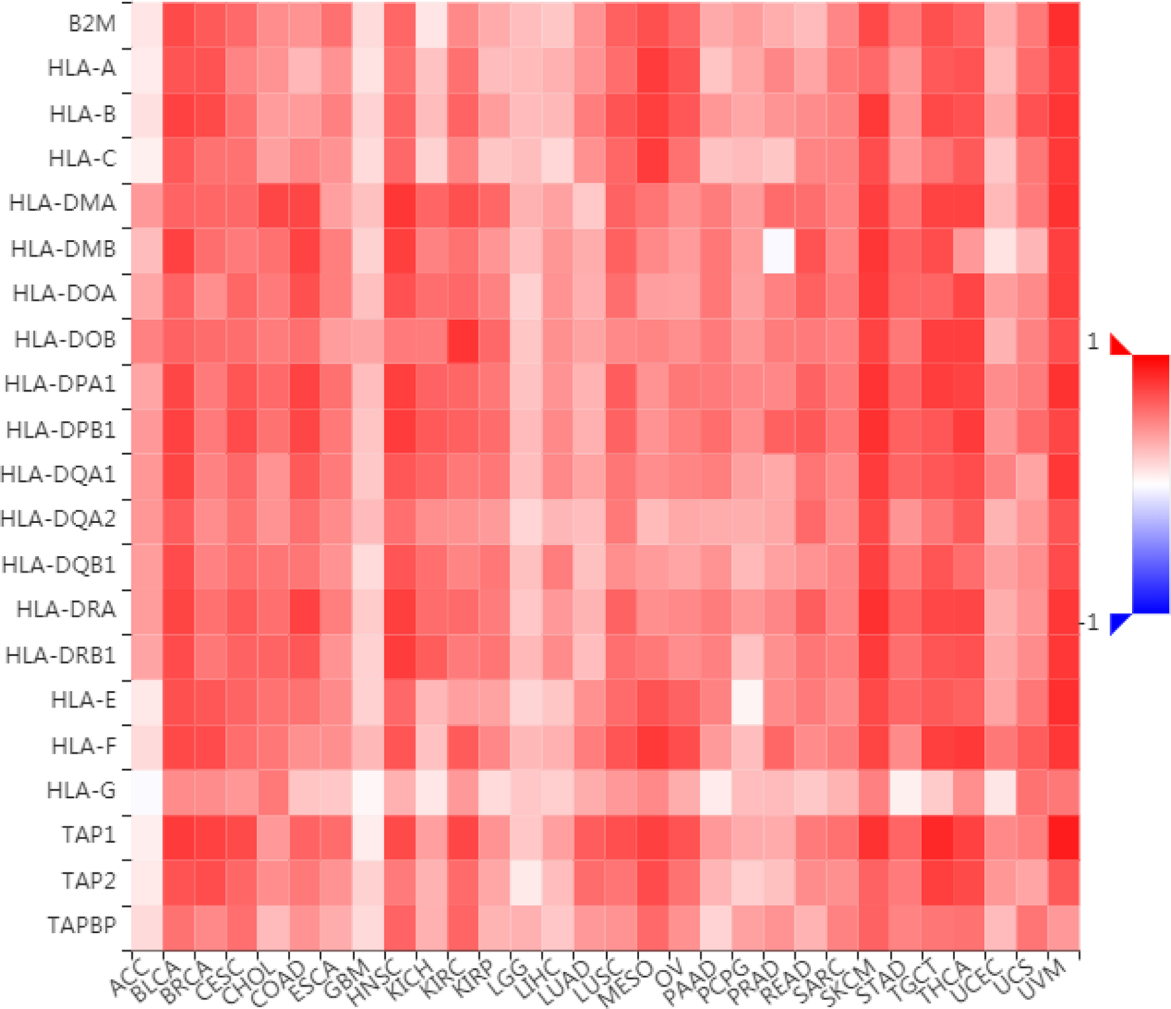
LAG3 expression is correlated with MHC molecules in pan-cancer.

To further understand the role of LAG3 in regulating the immune microenvironment of pancancer, we investigated the relationships between LAG3 expression and three types of previously described immune modulators (38) through the TISIDB database, an integrated repository portal for tumor-immune system interactions. Intriguingly, a similar correlation pattern between immune modulators and LAG3 was observed in 30 types of cancer, and most immunoinhibitors and immunostimulators positively correlated with LAG3 (**Figures 7**, **8**), although a minority of each negatively correlated with LAG3. More interestingly, we found that LAG3 positively correlated with most MHC molecules in pancancer (**Figure 9**). These findings suggest that LAG3 regulates the tumor immune microenvironment by synergizing with other immune modulators.

### LAG3 Synergizes With Other Checkpoint Members in the Tumor-Induced Immune Response

**Figure 10:**
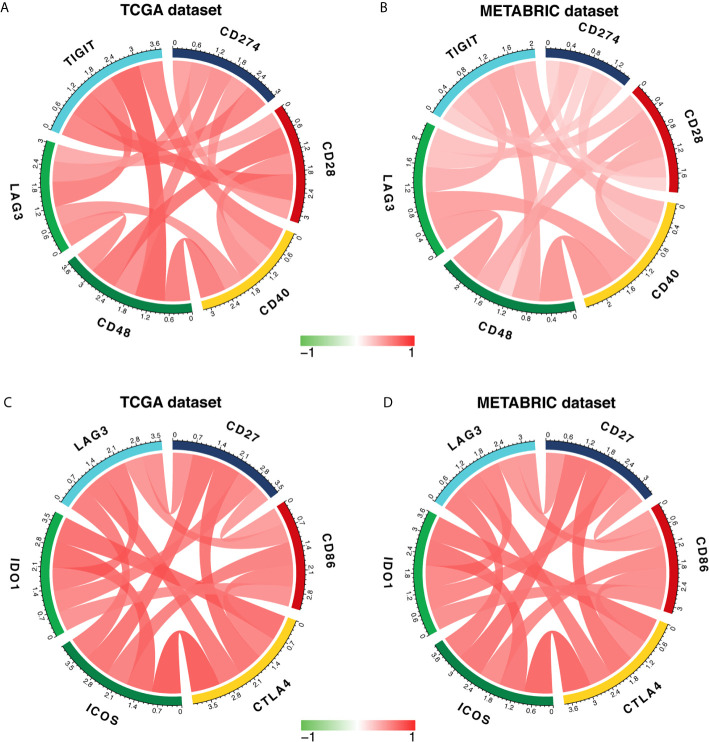
LAG3 expression is correlated with immune checkpoint members in TCGA and METABRIC databases **(A–D)**.

To further characterize the synergistic role of LAG3 in the breast cancer-induced immune response, we evaluated the correlations between LAG3 and other checkpoint members (**Figures 10A–D**). Strong correlations were observed between LAG3 and other checkpoint members. LAG3 positively correlated with TIGIT (r = 0.723, r = 0.465, TGCA, and METABRIC respectively), CD274 (PD-L1) (r = 0.592, r = 0.365), CD28 (r = 0.496, r = 0.364), CD40 (r = 0.742, r = 0.607), CD48 (r = 0.636, r = 0.652), and other checkpoint molecules including CD27 (r = 0.647, r = 0.594), CD86 (r = 0.614, r = 0.609), CTLA4 (r = 0.762, r = 0.722), ICOS (r = 0.754, r = 0.744), and IDO1 (r = 0.756, r = 0.696).

### The Relationship Between LAG3 and Specific Cellular Immune Responses

**Figure 11:**
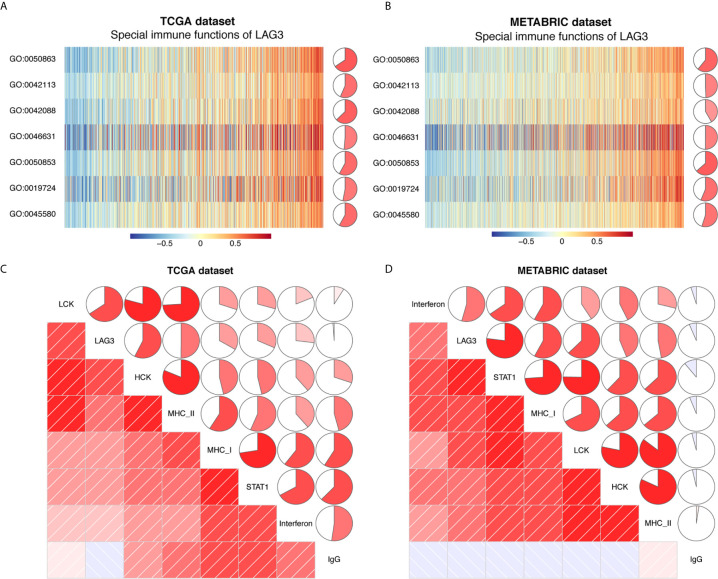
LAG3 related cell immunity and inflammatory activities in breast cancer. The relationship between LAG3 and cell immunity in TCGA and METABRIC datasets **(A, B)**. The relationship between LAG3 and inflammatory activities in TCGA and METABRIC datasets **(C, D)**. GO: 0019724: B cell mediated immunity; GO:0042088: T-helper 1 type immune response; GO:0042113: B cell activation; GO:0045580: regulation of T cell differentiation; GO:0046631: alpha-beta T cell activation; GO:0050853: B: cell receptor signaling pathway; GO:0050863: regulation of T cell activation.

Previous studies document the inhibitory role of T cell activation (12), although it is unclear whether LAG3 plays the same role in breast cancer and whether LAG3 influences other immune cells. To elucidate the relationship between LAG3 and specific immune responses in breast cancer, GSVA analysis was performed. Strong correlations between LAG3 and T and B cell immunity were observed (**Figures 11A, B**). LAG3 positively correlated with the T-helper 1 type immune response, regulation of T cell differentiation, regulation of T cell activation, and alpha-beta T cell activation. Furthermore, LAG3 positively correlated with B cell-mediated immunity, B cell activation, and B cell receptor signaling pathways. Moreover, these results were mutually validated using the TCGA and METABRIC databases. These findings suggest that LAG3 plays an inhibitory role in T cell-mediated tumor immunity in breast cancer, and likely affects B cell immunity.

### The Relationship Between LAG3 and Inflammatory Activities

To further reveal LAG3-related inflammatory activities, 104 genes derived from seven clusters were defined as metagenes using Gene Sets Variation Analysis (GSVA) (31) (**Table S1**) representing different types of inflammation and immunity. We found that LAG3 positively correlated with LCK, HCK, MHC-I, MHC-II, STAT1, and interferon, but not with IgG (**Figures 11C, D**). Among these seven clusters, LAG3 correlated most strongly with LCK metagenes. More important, these results were mutually verified using TCGA and METABRIC databases. These findings further suggest that LAG3 plays important immune and inflammatory functions in breast cancer.

## Discussion

As a novel therapeutic approach, immune checkpoint blocking therapy, which reactivates T cell immune responses to tumor cells and breaks tumor immune suppression, achieved marked success in preclinical or clinical trials of many malignant tumors (5, 7, 8, 11, 39). The most extensively used immune checkpoint inhibitors for research and application of cancer therapy include PD-1 and inhibitors of its ligand PD-L1, as well as CTLA-4. However, the objective response rates range between 13 and 56%, and complete response rates range between 1 and 16%, which presents frustrating challenges, particularly for breast cancer (5–11). Therefore, further progress on understanding the tumor microenvironment is urgently required to identify alternative or facilitating therapeutic targets.

Many recent studies show a specific correlation between LAG3 and PD-1 with T cell inhibition in various diseases (12, 40) such as in viral infection (12, 41), chronic tuberculosis (42), plasmodial infections (43), chronic lymphocytic leukemia (44), and ovarian cancer (45). However, the coexpression and effects of LAG3 and PD-1 on T cells in breast cancer patients are unclear. To define the molecular and clinical relationships between LAG3 expression and immune activities in breast cancer will greatly promote the identification and clinical application of a novel therapeutic target as well as to optimize current therapeutic strategies.

In the present study, we systematically analyzed the expression of LAG3 in breast cancer. We found that LAG3 was upregulated in breast cancer tissue, particularly enriched in the basal, HER2-positive, and LumA subtypes, as well as in patients with higher tumor grades. LAG3 therefore may serve as a valuable biomarker for the TNBC subtype. Moreover, previous studies show that the presence of LAG3+ intraepithelial tumor infiltrating lymphocytes (iTILs) is significantly related to younger age, large tumor size, ER/PR-negativity, and a high Ki67 proliferation index (46, 47). Together, these results indicate that high expression of LAG3 predicts a highly malignant breast cancer. However, some studies arrived at the seemingly opposite conclusion that high expression of LAG3 is associated with favorable overall survival of patients with solid tumors including ovarian, gastric, lymphoma, NSCLC, colorectal, and renal (48), as well as breast cancer. We therefore must focus on the important immunological role of LAG3.

LAG3 is likely predominantly expressed in immune cell populations in the tumor microenvironment, but not by breast cancer cells (12, 49). Under physiological conditions, LAG3, which is expressed on the membranes of activated human T cells, NK cells, B cells, and DCs (50–53), is an activation marker for CD4+ and CD8+ T cells. In tumor patients, LAG3 is expressed on surface of TILs (54, 55). Early studies suggest that LAG3 is a negative regulator of T-cell activation, and the regulation of T cell-mediated immune responses mainly involve three aspects as follows.

First, the proliferation and activation of T cells is directly inhibited by negative regulation. Previous studies show that LAG3 is a negative regulator of T-cell activation, and blockade of LAG3 function in human CD4 clones enhances cell proliferation with elevated production of IFN-γ, TNFα, IL-2, and IL-4 (16). Furthermore, the highly conserved motif KIEELE mediates a cell-intrinsic signal, which may be essential for the negative regulatory function of LAG3 on T cells (56). A more specific role for LAG3 on CD8+ T cells was demonstrated using a model of self-tolerance. Thus, adoptively transferred LAG3−/− HA-specific CD8+ T cells were expanded and produced large amounts of IFNγ, indicating that LAG3 limits self-tolerance (52). Moreover, these CD8+ T cells regain effector function, indicated by an increased number of IFNγ-producing cells. Therefore, the hypothesis is not dependent on CD4+ T cells, and the effect induced by blocking LAG3 is a CD8+ T-cell intrinsic effect (52).

Second, the T cell immune response is indirectly suppressed by promoting the inhibitory function of regulatory T cells (Treg). Recent studies show that LAG3 promotes the differentiation of Tregs, while its blockade inhibits the induction of Tregs (57). This study further illustrates that CD4+ T cells are skewed into a Th1 phenotype by blockade or genetic deletion of LAG3, with LAG3 limiting IL-2 and STAT5 signaling that modulates the ability to be suppressed by Tregs.

Third, T cell activation is prevented by regulating antigen-presenting cells (APC) (14), which is supported by our finding that LAG3 closely correlated with antigen processing and presentation pathways. Published studies show that LAG3 may be involved in mediating bidirectional signaling between interacting APCs. DC activation is inhibited by MHC class-II binding to LAG3-expressing Tregs to suppress their maturation (58). Interestingly, previous studies focus on the impact of LAG3 on T cell immunity, although whether LAG3 impacts other immune response and immune cell populations is unclear. Here we found that LAG3 positively correlated with B cell-mediated immunity, B cell activation, B cell receptor signaling pathways, and natural killer cell-mediated cytotoxicity pathways. Consistent with our observations, previous studies indicate that LAG3 expression is related to NK cells and activated B cells in a T cell-dependent manner (59).

We observed that LAG3 expression had the strongest correlation with T cells (particularly CD8+ T cells), followed by plasmacytoid dendritic cells, NK cells, the monocytic lineage, and the B lineage. LAG3 is constitutively expressed by plasmacytoid dendritic cells (pDCs) at a much higher level than any other cell type (60), while LAG3 is not expressed by any lymphoid DC or myeloid subset. Compared with wild-type pDCs, LAG3–pDCs show enhanced expansion following CpG stimulation *in vivo*, but do not have an altered expression profile of activation markers, including differential cytokine production or CD80/86 and MHC class II molecules (60).

Furthermore, LAG3+ pDCs are involved in the melanoma environment and interact with HLA-DR-expressing tumor cells *in vivo*. Moreover, *in vitro* studies show that as the result of the stimulation of MHC class II-expressing melanoma cells, LAG3+ pDCs mature and produce IL-6 (61). Therefore, LAG3+ pDCs may indirectly drive myeloid-derived suppressor cell (MDSCs)-mediated immunosuppression through the engagement of MHC class II+ melanoma cells.

LAG3 is expressed by NK cells (~10%) and invariant NKT cells (19). LAG3 signaling reduces the proliferation of activated NKT cells, resulting in cell cycle arrest in S (62). Moreover, overexpression of LAG3 is associated with impaired iNKT cytokine production (IFNγ) during chronic HIV infection, although this does not involve other T-cell subsets (63). One study suggests that a soluble monomeric form of LAG3 (sLAG3), generated by alternative splicing, impairs the differentiation of monocytes into DCs and macrophages, which subsequently diminishes its immunostimulatory capacity (64). Moreover, at the end of IMP321 (a LAG3 antagonist) treatment, there is a 50% objective tumor response and decreased tumor size related to an increase in the absolute number of monocytic cells (65).

The role of LAG3 on B cells is partially understood, because its expression is only reported in a single study (43). Thus, LAG3 exerts differential inhibitory impacts on various types of lymphocytes. Except for relatively deep and detailed research on T cells, the functional role and mechanism of LAG3 on other immune cells are not fully understood, and further studies are required to enrich this field.

As described above, LAG3 expression was associated with poor clinicopathological factors and elicited an immune suppressive function, supporting the hypothesis that the expression of LAG3 in breast cancer patients leads to poor survival. However, inconsistent with the present results, the findings of other studies indicate a favorable association between high expression of LAG3 and cancer-specific survival, particularly of the ER-negative, HER2-positive, and basal-like subtypes (46, 66, 67). Interestingly, another study found that serum LAG3 closely correlates with prolonged survival of ER-positive patients (41).

These results indicate a complicated relationship between LAG3 expression, clinical characteristics, and the prognosis of breast cancer. One possible explanation is that the presence of LAG-3 expressing TILs may indicate an ongoing cancer-immune interaction (46), a phenotype defined as an inflamed tumor (68), which usually signifies somewhat improved prognosis. Furthermore, LAG3 expression by engineered tumor cells efficiently promotes and facilities activation, intratumoral recruitment, and Th1 commitment of APCs, which results in a large intratumoral influx of specific and non-specific reactive cells, as well as the release of immunoregulatory and cytotoxic mediators (69). Consequently, further studies are encouraged to focus on this controversial problem.

Despite the promising impact of cancer immunotherapy targeting CTLA4 (e.g. ipilimumab) and PD1/PDL1 (e.g. pembrolizumab), with in-depth research, the side effects and resistance of these drugs have gradually emerged (70, 71). Moreover, a large number of cancer patients fail to respond, and the response rate to ipilimumab is only 15% and that to PD-1/PD-L1 inhibitors is <40% (72). Here we found that LAG3 closely correlated with PD-L1 expression and the PD-1 checkpoint pathway in cancer; and strong correlations were observed between LAG3 and other checkpoint members such as CTLA4, TIGIT, CD28, CD40, CD48, CD27, CD86, ICOS, and IDO1.

Early studies found that sustained T-cell activation induced by a chronic inflammatory environment, for example, during chronic viral infection or in a tumor, causes persistent LAG3 expression by T cells, which frequently coexpresses with other IRs such as PD1, TIM3, TIGIT, CD160, and 2B4, subsequently resulting in a dysfunctional T-cell state (73). This state, named T-cell functional exhaustion, is defined by a distinct subset of exhausted T cells with elevated expression of IRs, resulting in lack of proliferation, cytokine secretion, and cytolytic activity (15, 74–76).

Here we characterized the comprehensive pattern of LAG3 expression and immune cell populations and immune modulators. These results should be considered in the context of limitations, and future studies must analyze or discuss related specific immune cells and immune molecules related to enhancing the significance of the application of these results.

## Data Availability Statement

The original contributions presented in the study are included in the article/**Supplementary Material**. Further inquiries can be directed to the corresponding authors.

## Ethics Statement

All procedures performed in studies involving human participants were in accordance with the ethical standards of the institutional and/or national research committee and with the 1964 Helsinki declaration and its later amendments or comparable ethical standards.

## Funding 

This work was partially sponsored by grants from, National Key R&D Program of China (Grant No. 2018YFC1312100), the National Natural Science Foundation of China, No. 81872160 (JW), Beijing Municipal Natural Science Foundation (Key program, No. 7191009) (JW), Capital Public Health Education, Beijing Science and Technology Program (No. Z161100000116093) (JW) and CAMS Initiative for Innovative Medicine (No. 2016-12M-1-007) (JW). This article was partially supported by PUMC Youth Fund and the Fundamental Research Funds for the Central Universities (No. 3332015157) (YF), as well as Capital Public Health Education, Beijing Science and Technology Program (No. Z171100000417028) (YF).

## Authors Contributions

Conception and design: JW, YF, ZW, and QL. Development of methodology: QL and YQ. Acquisition of data: JZ, XK, and XW. Analysis and interpretation of data: QL and JZ. Writing, review and/or revision of the manuscript: QL, YQ, and JZ. Administrative, technical, or material support: XK and XW. Study supervision: JW, YF, and ZW. All authors contributed to the article and approved the submitted version.

## Conflict of Interest

The authors declare that the research was conducted in the absence of any commercial or financial relationships that could be construed as a potential conflict of interest.

## Supplementary Material

Supplementary Figure 1 LAG3 serves as a potential biomarker. LAG3 expression pattern between TNBC and non-TNBC tissues in TCGA and METABRIC **(A, B)**; ROC curves predicted LAG3 as a biomarker of TNBC **(C, D)**. (*P < 0.05, **P < 0.01, ***P < 0.001, ***P < 0.0001).

Supplementary Table 1 Detailed gene list of seven clusters of metagenes.
