# Supplementary figures and images for: Molecular and Clinical Characterization of LAG3 in Breast Cancer Through 2994 Samples
Source: Front Immunol. 2021 Jun 29;12:599207. doi: 10.3389/fimmu.2021.599207 (PMC8276078; doi:10.3389/fimmu.2021.599207)

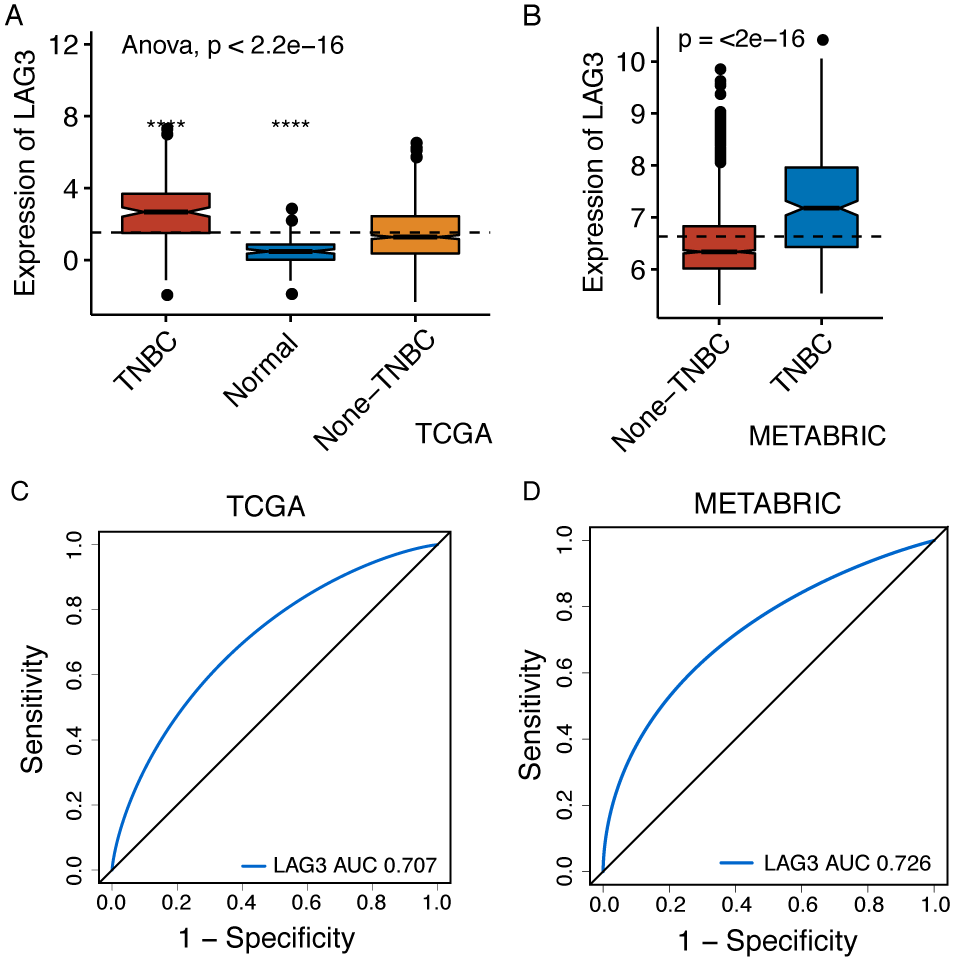

Supplement: Supplementary Figure 1 — LAG3 serves as a potential biomarker. LAG3 expression pattern between TNBC and non-TNBC tissues in TCGA and METABRIC (A, B); ROC curves predicted LAG3 as a biomarker of TNBC (C, D). (*P < 0.05, **P < 0.01, ***P < 0.001, ***P < 0.0001). [file Image_1.tif]
