# Supplementary material for: Molecular and Clinical Characterization of LAG3 in Breast Cancer Through 2994 Samples
Source: Front Immunol. 2021 Jun 29;12:599207. doi: 10.3389/fimmu.2021.599207 (PMC8276078; doi:10.3389/fimmu.2021.599207)
Supplement: Supplementary Table 1 — Detailed gene list of seven clusters of metagenes. [file Table_1.pdf]

HCK	IgG	Interferon	LCK	MHC_I	MHC_II	STAT1
C1QB	IGSF8	IFIT1	CD2	HLA-E	HLA-DRB	TAP1
C1QA	ISLR2	IFIT3	GZMK	HLA-H	HLA-DRB	STAT1
AIF1	IGSF21	IFI44L	GZMA	HLA-B	HLA-DRB	CXCL10
LST1	IGSF1	OAS3	CD3D	HLA-J	HLA-DPA	CXCL11
DOCK2	IGSF22	MX1	CD53	HLA-F	HLA-DRA	GBP1
LAPTM5	IGDCC3	RSAD2	LCK	HLA-G	HLA-DQA	CXCL9
TYROBP	IGHD	IFI44	ARHGAP1	HLA-A	HLA-DQA2	
MS4A4A	IGSF11	OAS2	CCL5	HLA-C	HLA-DMA	
MS4A6A	IGSF5	OAS1	GMFG	HLA-L	HLA-DOA	
CD163	IGSF6		SELL		HLA-DRB4	
ITGB2			STAT4		HLA-DMB	
SLC7A7			SAMSN1		HLA-DQB1	
LAIR1			RAC2		HLA-DPB1	
HCK			HCLS1		HLA-DQB2	
TFEC			CCR7		CD74	
IFI30			PIK3CD		PTPRC	
MNDA			CORO1A		HLA-DOB	
FCER1G			CD48		HLA-DPB2	
RNASE6			IL2RG			
SLCO2B1			SH2D1A			
CCR1			SLAMF1			
			IL7R			
			INPP5D			
			KLRK1			
			FGL2			
			IRF8			
			SELPLG			
			IL10RA			
			SLA			
			CCR2			
			CSF2RB			
